# Retraction Note: Theoretical Limits of Energy Density in Silicon-Carbon Composite Anode Based Lithium Ion Batteries

**DOI:** 10.1038/s41598-019-51047-6

**Published:** 2019-11-04

**Authors:** Ranjan Dash, Sreekanth Pannala

**Affiliations:** 1 SABIC, 475 Creamery Way, Exton, PA 19341 USA; 2 SABIC, 14100 Southwest Freeway, Sugar Land, TX 77478 USA

Retraction of: *Scientific Reports* 10.1038/srep27449, published online 17 June 2016

The editors retract this article.

Equation (3) in the article, describing the porosity of the anode, contains a normalization error. Instead of using the volume fractions of silicon and graphite in the calculation of the anode porosity, their weight fractions are used. When the relationship between the volumetric capacity of the anode and the silicon amount (Figure 2 in the article) is correctly calculated, this relationship does no longer have a maximum: the volumetric capacity continuously increases with the increasing silicon amount in the anode. The relationships between volumetric and gravimetric capacities of electrodes made with different materials, shown in Figure 3, no longer hold. Since the conclusions of the article are based on the presence of the threshold value of Si amount in the electrode, which is incorrect, the editors retract the article.

The authors disagree with the retraction.

